# Findings From the Step Up, Test Up Study of an Electronic Screening and Brief Intervention for Alcohol Misuse in Adolescents and Young Adults Presenting for HIV Testing: Randomized Controlled Efficacy Trial

**DOI:** 10.2196/43653

**Published:** 2023-03-29

**Authors:** Niranjan S Karnik, Lisa M Kuhns, Anna L Hotton, Natascha Del Vecchio, Moira McNulty, John Schneider, Geri Donenberg, Kristin Keglovitz Baker, Rose Diskin, Abigail Muldoon, Juan Rivera, Faith Summersett Williams, Robert Garofalo

**Affiliations:** 1 Institute for Juvenile Research Department of Psychiatry University of Illinois Chicago Chicago, IL United States; 2 The Potocsnak Family Division of Adolescent and Young Adult Medicine Ann & Robert H Lurie Children's Hospital of Chicago Chicago, IL United States; 3 The Chicago Center for HIV Elimination The University of Chicago Chicago, IL United States; 4 Center for Dissemination and Implementation Science University of Illinois Chicago Chicago, IL United States; 5 Center for Education, Research & Advocacy Howard Brown Health Chicago, IL United States

**Keywords:** HIV prevention, men who have sex with men, transgender women, alcohol intervention, HIV, gay, homosexual, MSM, alcohol, youth, screening, sexual behavior, sexual behavior, sexual risk, risky, pre-exposure prophylaxis, prophylaxis, prevention, efficacy, adolescent, young adult, testing, risk

## Abstract

**Background:**

Substance use, particularly binge drinking of alcohol and noninjection substance use, is associated with increased risk for HIV infection among youth, but structured substance use screening and brief intervention are not often provided as part of HIV risk reduction.

**Objective:**

The purpose of the study was to test the efficacy of a fully automated electronic screening and brief intervention, called Step Up, Test Up, to reduce alcohol misuse among adolescents and young adults presenting for HIV testing. Secondary objectives were reduction in sexual risk and uptake of pre-exposure prophylaxis (PrEP) for HIV prevention.

**Methods:**

Youth aged 16 years to 25 years who presented for HIV testing at community-based locations were recruited for study participation. Those who screened at moderate to high risk on the Alcohol Use Disorders Identification Test were randomized (1:1) to either an electronic brief intervention or a time-attention control. The primary outcome was change in alcohol use at 1, 3, 6, and 12-month follow-ups. Negative binomial and log binomial regression analyses with generalized estimating equations were conducted to evaluate the intervention efficacy.

**Results:**

Among a sample of 329 youth, there were no significant differences in alcohol use outcomes between conditions over time or at the 1, 3, 6, or 12-month time points. In terms of secondary outcomes, there was evidence of reduction in condomless insertive anal sex under the influence of alcohol and drugs at 12 months compared with 3 months in the intervention versus the attention control condition (incidence rate ratio=0.15, 95% CI 0.05-0.44); however, there were no other significant differences in sexual risk and no difference in PrEP engagement.

**Conclusions:**

We found no effect of electronic brief intervention to reduce alcohol use and some effect on sexual risk among youth aged 16 years to 25 years who present for HIV testing.

**Trial Registration:**

ClinicalTrials.gov number NCT02703116; https://clinicaltrials.gov/ct2/show/NCT02703116

**International Registered Report Identifier (IRRID):**

RR2-10.1186/s12889-020-8154-6

## Introduction

The syndemic intersection of HIV risk and substance misuse among young men who have sex with men (YMSM) and young transgender women (YTW) has been well established and well documented [1-5]. Recent studies have shown that substance use, particularly binge drinking of alcohol and noninjection substance use, increases sexual risks and the potential for HIV infection [6]. In 2019, over 20% of new HIV infections occurred in youth aged 13 years to 19 years [7]. Among young people, the vast majority (66%) of HIV infections continue to occur among those having male-to-male sexual contact with YMSM constituting the largest share. Among those with male-to-male sexual contact, 25% of infections occur among YMSM aged 13 years to 24 years, and 31% occur among those aged 25 years to 29 years [7]. In addition to YMSM, substantial evidence exists that YTW are also at risk for HIV, and in 2019, their rate of diagnosis increased by 5% [7]. Along with routine condom use and pre-exposure prophylaxis (PrEP), addressing alcohol and other drug (AOD) use are among the most modifiable individual-level factors for the prevention of HIV and sexually transmitted infections (STIs). Although there are many structural factors including racism, stigma, and poverty that contribute to the dynamics of HIV infection, prevention strategies have often focused on individual or personal-level factors. Recent years have seen shifts toward HIV interventions in community clinics that provide primary care or specialized HIV services. At the intersection of individual behavioral change and structural change are attempts to insert brief motivational strategies into larger systems of care. Motivational interviewing (MI)–based approaches promote screening, brief intervention, and referral to treatment (SBIRT). Electronic screening and brief intervention (eSBI) is a subset of SBIRT in an electronic medium suitable for use in primary care and other generalist settings. YMSM and YTW often seek HIV testing and other supportive services in community-based and outreach settings. These settings are underutilized as potential entry points for engagement in comprehensive care across the HIV prevention and care continuum, including PrEP for HIV-negative youth who are at risk of HIV acquisition. We theorized that these same community settings could serve as an access point for substance use interventions if these interventions were brief and scalable.

The purpose of this study was to assess the feasibility, acceptability, and initial efficacy of eSBI in comparison to an electronic attention control intervention (ie, promotion of good nutrition), coupled with standard HIV prevention, on alcohol use among YMSM and YTW in community-based HIV testing environments in Chicago. Secondary objectives were to assess intervention effects on sexual behavior, as well as engagement within the HIV prevention and care continuum, and to assess modification of the intervention effect by comorbid mental health problems (ie, symptoms of depression and anxiety).

## Methods

### Design

**Figure 1:**
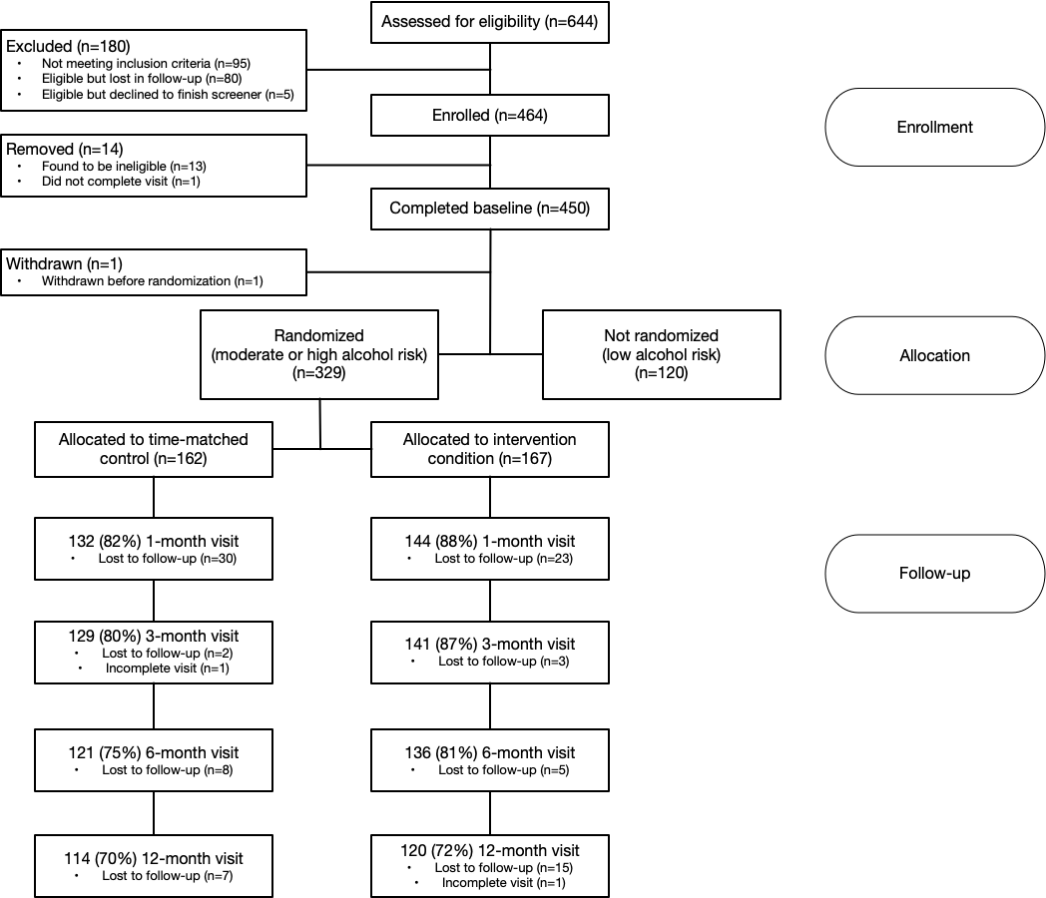
Step up, Test Up CONSORT diagram.

This study was a randomized controlled trial of eSBI among YMSM and YTW seeking HIV testing [8]. Using an electronic web-based portal, all participants were screened for alcohol misuse and received immediate feedback regarding their level of use (eg, how their use compares to others, whether it exceeds “safe use” guidelines); those who screened for moderate to high alcohol use, including binge drinking, were then randomized to either electronic intervention or control modules. All participants, regardless of randomization status, were followed for 12 months with in-person visits conducted at 1, 3, 6, and 12-month intervals (see CONSORT diagram, Figure 1). All participants were recruited from HIV testing clinics that utilized a seek, test, treat, and retain (STTR) model of care [9].

### Ethical Approval

The protocol was approved by the Ann & Robert H. Lurie Children’s Hospital Institutional Review Board (review #2015-703) with a waiver of parental permission for participation of minors (aged 16-17 years). All enrolled participants were consented prior to the start of any research activities using an electronic consent form. The consent form indicated that the purpose of the study was to learn more about whether screening and brief intervention for substance use is effective in reducing that use.

### Identification and Recruitment of Participants

Youth were recruited from HIV testing centers at the 3 primary sites in Chicago: the Division of Adolescent Medicine at Lurie Children’s Hospital, Howard Brown Health, and the Village at the University of Chicago. Individuals were eligible if they were (1) aged 16 years to 25 years, (2) interested in testing for HIV infection, (3) HIV-negative or HIV status unknown (per self-report; verified at the point of HIV testing), (4) identified as a man who has sex with men or a transgender woman who has sex with men (ie, born male, identify as female or transgender, and at any point in the gender transition process), and (5) English-speaking. Individuals who were eligible and interested were enrolled by study staff.

### Randomization

Upon enrollment, all participants completed HIV testing (using standard of preventive care testing and counseling at each site); individuals who screened reactive on the rapid HIV test were immediately referred for confirmatory HIV testing and were withdrawn from the study. All participants were screened for alcohol misuse using the Alcohol Use Disorders Identification Test (AUDIT) via an electronic portal. The AUDIT is a widely used, 10-item, validated screening tool for the assessment of alcohol use [10]. It contains items that reflect consumption, dependency, and alcohol-related harms. The AUDIT total score ranges from 0 to 40, divided into 4 Zones (I-IV), reflecting increasing levels of risk and recommended intervention (ie, Zone 1: alcohol education; Zone II: simple advice; Zone III: simple advice plus brief counseling and monitoring; Zone IV: referral to specialist for diagnostic evaluation and treatment). In addition, one item on the AUDIT reflects binge drinking (“How often do you have six or more drinks on one occasion?”). For our study, those scoring in Zones II-IV or endorsing the binge drinking item were included.

After completion of the AUDIT, participants received immediate feedback regarding their level of use. Participants who screened moderate to high risk for alcohol misuse or endorsed binge drinking on the AUDIT were randomized via computerized assignment (1:1) to either brief intervention modules to reduce alcohol misuse, eSBI, or a time and attention-matched control (ie, promotion of good nutrition). Study staff were blinded to random assignments. The randomization sequence was generated by NSK, uploaded to the Radiant platform (Radiant Interactive Group), and assigned automatically in the order of enrollment.

### Description of the Intervention: eSBI

**Figure 2:**
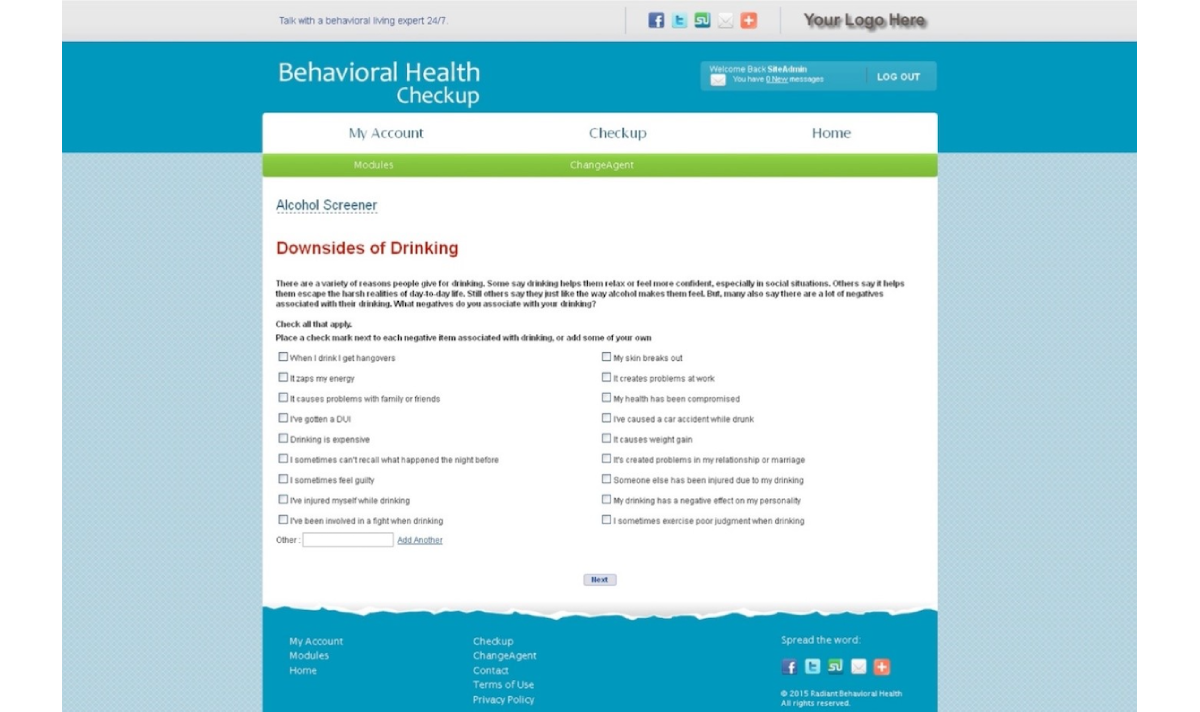
Example slide from the electronic screening and brief intervention (eSBI).

Those randomized to the intervention were asked to complete lessons or exercises on 11 topical areas focused on alcohol use (eg, importance of change, downsides of drinking, barriers to change, strategies for cutting back, change motivators) in an MI format, each with a single web page. The intervention is not a full MI intervention, as it was relatively simple in its presentation, but it did have components of the Elicit-Provide-Elicit approach. The intervention had been widely used in employee assistance programs. The intervention content was adapted for late adolescents and young adults in terms of voice and language (eg, open, nonjudgmental, inclusive), contexts (eg, school as well as work contexts), and reading level. The intervention was presented as a series of slides that contained an interactive element in which participants could click on checkboxes, drop-down menus, and short text fields as part of the brief MI exercises. An example slide is shown in Figure 2. By design, the intervention lacked any specific cultural tailoring for YMSM or YTW other than the age-appropriate language. This was intended to facilitate rapid scaling and broad adoption across the general population if the intervention proved effective. The electronic intervention and control modules were delivered using a customized eSBI platform developed by Radiant Interactive Group (Laguna Niguel, CA). Radiant uses industry-standard data encryption and security to manage protected health information recorded by their system.

### Time and Attention-Matched Control: Diet and Nutrition

Those randomized to the control condition completed a brief time and attention-control intervention, also in an MI format and of equal length (ie, same number of intervention screens), which encouraged good nutrition but was not expected to impact alcohol use.

### Standard of Care Prevention Services

The standard HIV preventive care at each site included rapid point-of-care HIV testing and counseling and linkage navigation to PrEP or HIV care, depending on the test result.

### Study Assessments

Participants completed a baseline study visit that included a standardized assessment via computer-assisted self-interviewing (CASI). Follow-up CASI assessments were conducted at 1, 3, 6, and 12-month follow-up visits either in person or remotely via web-based assessment. Detailed contact information was collected to facilitate study retention.

#### Primary Outcome

All outcome measures were self-reported and constructed using the revised versions of the Daily Drinking Questionnaire [11] (DDQ-R [12]) including questions regarding the heaviest drinking week [13]. The questionnaire asks the participant to report the number of drinks consumed for each day of the week for both a typical week and heavy drinking week in the past 30 days. The outcomes assessed for this analysis were (1) the total number of drinks consumed in a typical drinking week, (2) the total number of drinks consumed in a peak drinking week, (3) the number of days 5 or more drinks were consumed in a typical week, (4) the number of days 5 or more drinks were consumed in a heavy drinking week, and (5) any binge drinking (ie, 5 or more drinks consumed in 1 episode) in the prior month.

#### Secondary Outcomes

Sexual risk behavior was measured via a modified version of the AIDS-Risk Behavior Assessment [14] used in prior studies with YMSM and YTW [15,16] and included (1) condomless receptive anal sex (RAS) acts, (2) condomless RAS acts while under the influence of alcohol or drugs, (3) condomless insertive anal sex (IAS) acts, and (4) condomless IAS acts while under the influence of alcohol or drugs. Condomless sex was defined as sex in which “no condom was used or a condom was used but only for a part of the time.” Attending at least one provider visit for PrEP care during the observation period was additionally included as a secondary outcome for HIV-negative participants who reported using PrEP (n=89).

We measured eSBI intervention satisfaction and acceptability using the Client Satisfaction Questionnaire (CSQ-8) [17]. Participants were asked to rate the quality of the intervention and their satisfaction, as well as whether they would recommend it to friends. We also include an open-ended item, “Do you have any other suggestions to improve this intervention?”

#### Moderators

Depression and anxiety were measured using the short version of the Center for Epidemiologic Studies Depression Scale (CES-D10) [18] and 7-item Generalized Anxiety Disorder (GAD-7) [19], respectively.

### Statistical Analysis

The analysis used an intent-to-treat approach in which all randomized participants were included for analysis. Baseline characteristics of the intervention groups were inspected visually for imbalances; differences between the 2 intervention groups were assessed using chi-square or Fisher exact tests for categorical variables and *t* tests for numerical variables. Changes in drinking levels over time were examined descriptively using means with SDs and medians with IQRs. The targeted sample size of 450 was estimated to yield 80% power to detect effects for our primary (alcohol use) outcome under a range of retention levels (75%-85%).

To evaluate the efficacy of the intervention, generalized estimating equations (GEEs) with robust standard errors were conducted. Each outcome was regressed on intervention group, time, a group-by-time interaction, and the baseline value of the outcome, where the statistical significance of the interaction term indicates whether the groups differ in response to the intervention over time. The baseline outcomes were included in the model due to baseline imbalances in the outcomes between the groups. Unadjusted models are presented in Table S1 in Multimedia Appendix 1. Omnibus tests (Type III Score tests) were used to assess the overall effects of the intervention, time, and whether the differences between the groups varied across time points. Contrasts were performed to assess the effect of the intervention compared with the time and attention-matched control at each follow-up visit compared with first follow-up for exploratory purposes even when omnibus tests were not significant. Interaction contrasts represent the relative risk (RR) for binary outcomes or incidence rate ratio (IRR) for count outcomes in the intervention arm at that time point compared with the RR or IRR for the group comparison at first follow-up (ie, a ratio of ratios). All count outcomes were modeled as negative binomially distributed variables with log link functions to compute rate ratios; the binary outcome (having had more than 5 drinks in any single drinking session in the prior month) was modeled as a binomially distributed variable with log link function to estimate relative risk. An exchangeable working correlation was specified to account for repeated measurements on participants over time. All statistical analyses were conducted using SAS software version 9.4 (SAS Institute). Graphics were generated using STATA 17.

## Results

### Participants

A total of 644 individuals were screened for the study: 549 were eligible, and 95 were not eligible. Study enrollment began in December 2016, and all follow-up visits were completed by October 2020. Among those eligible, 85 (85/549, 15.5%) were lost to follow-up prior to enrollment, resulting in 464 enrolled (see CONSORT diagram, Figure 1). Of those enrolled, 14 were withdrawn or considered incomplete, resulting in a final sample of 450 (450/549, 82% of eligible).

All enrolled participants were screened with the AUDIT; 120 screened low risk and were not randomized. Therefore, a total of 329 participants were included in the analysis described herein. Table 1 presents descriptive information on the 329 participants randomized to the intervention or attention control conditions. The average age of the participants was 22.8 years. Most participants identified as non-White (221/329, 67.2%). The majority identified as cisgender male (294/329, 89.4%) and gay (205/329, 62.3%). Most of the participants were employed either full-time or part-time (222/329, 67.7%) and had at least some college education or attended trade school (224/329, 68.3%). There were 70 (70/329, 21.3%) with a history of incarceration. A little more than one-half of participants had a primary care provider (174/329, 52.9%). There were no significant differences between the intervention groups in these characteristics at baseline. For each measure of drinking, there were reductions in the mean amounts of drinking over time in both groups (Table 2). The average duration of the eSBI intervention was just under 10 minutes and of the control condition was just over 10 minutes.

**Table 1:** Demographics for the intervention (electronic screening and brief intervention [eSBI]; n=167) and control (n=162) groups.

Characteristics			eSBI^a^		Control^a^		*P* value
Age (years), mean (SD)			22.89 (1.99)		22.79 (1.97)		.65
**Gender identity, n (%)**							.88
	Cisgender male	150 (89.8)		144 (88.9)			
	Transgender female	15 (9.0)		15 (9.3)			
	Transgender male	2 (1.2)		3 (1.9)			
**Sexual orientation, n (%) **							.98
	Bisexual	42 (25.1)		41 (25.3)			
	Gay	105 (62.9)		100 (61.7)			
	Queer	16 (9.6)		16 (9.9)			
	Straight/heterosexual	4 (2.4)		5 (3.1)			
**Race/ethnicity, n (%)**							.68
	White NH^b^	60 (35.9)		48 (29.6)			
	Black/African American NH	65 (38.9)		68 (42.0)			
	Hispanic/Latino/a	24 (14.4)		26 (16.0)			
	Other NH	18 (10.8)		20 (12.3)			
**Employment, n (%)**							.44
	Employed full-time	54 (32.3)		63 (39.1)			
	Employed part-time	56 (33.5)		49 (30.4)			
	Unemployed	57 (34.1)		49 (30.4)			
**Education, n (%)**							.59
	Less than high school	15 (9.0)		13 (8.1)			
	High school or GED^c^	36 (21.6)		40 (24.8)			
	Some college or trade school	50 (29.9)		55 (34.2)			
	College degree or higher	66 (39.5)		53 (32.9)			
Arrest history, n (%)			44 (26.3)		53 (32.9)		.24
Incarceration history, n (%)			30 (18.0)		40 (24.8)		.17
Currently has a PCP^d^, n (%)			82 (49.1)		92 (56.8)		.20
**Baseline HIV test results, n (%)**							.21
	Nonreactive	167 (100.0)		159 (98.1)			
	Reactive	0 (0)		2 (1.2)			
	Confirmed positive	0 (0)		1 (0.6)			

^a^Not all sum to 329 due to missing data.

^b^NH: non-Hispanic.

^c^GED: General Education Development.

^d^PCP: primary care physician.

**Table 2:** Outcomes by treatment condition at all time points.

Outcomes			All participants (n=329)		Control (n=162)		eSBI^a^ intervention (n=167)
**Number of drinks in a typical drinking week, mean (SD)**							
	Baseline	11.89 (11.64)		10.51 (10.45)		13.24 (12.57)	
	1 month	8.92 (10.01)		9.33 (12.31)		8.55 (7.31)	
	3 months	8.51 (9.25)		7.94 (8.43)		9.04 (9.94)	
	6 months	7.90 (8.00)		7.49 (7.91)		8.27 (8.09)	
	12 months	8.68 (9.61)		8.35 (7.74)		8.98 (11.12)	
**Number of drinks in a typical drinking week, median (IQR)**							
	Baseline	8.00 (4.00-16.00)		8.00 (4.00-14.00)		10.00 (5.00-17.00)	
	1 month	6.00 (3.00-12.00)		6.00 (3.00-12.00)		7.00 (3.00-13.00)	
	3 months	6.00 (3.00-11.00)		6.00 (3.00-10.00)		6.00 (2.00-13.00)	
	6 months	6.00 (2.00-12.00)		5.00 (2.00-11.00)		6.00 (2.00-12.00)	
	12 months	7.00 (2.00-12.00)		7.00 (2.00-11.75)		6.00 (2.00-12.00)	
**Number of drinks in a peak drinking week, mean (SD)**							
	Baseline	6.36 (4.84)		5.83 (4.48)		6.87 (5.13)	
	1 month	5.09 (3.84)		5.15 (4.14)		5.04 (3.57)	
	3 months	4.96 (4.00)		4.71 (3.81)		5.18 (4.17)	
	6 months	4.86 (3.54)		4.62 (3.42)		5.08 (3.64)	
	12 months	4.90 (3.63)		4.75 (3.24)		5.04 (3.98)	
**Number of drinks in a peak drinking week, median (IQR)**							
	Baseline	5.00 (3.00-8.00)		5.00 (3.00-8.00)		6.00 (4.00-9.00)	
	1 month	5.00 (2.00-7.00)		4.00 (2.75-7.00)		5.00 (2.00-7.00)	
	3 months	4.00 (2.00-7.00)		4.00 (2.00-6.00)		4.00 (2.00-8.00)	
	6 months	4.00 (2.00-7.00)		4.00 (2.00-6.00)		4.50 (2.00-7.25)	
	12 months	5.00 (2.00-7.00)		5.00 (2.00-7.00)		5.00 (2.00-7.00)	
**Number of days with 5 or more drinks in a typical drinking week, mean (SD)**							
	Baseline	0.95 (1.41)		0.84 (1.30)		1.05 (1.51)	
	1 month	0.63 (1.14)		0.69 (1.24)		0.58 (1.03)	
	3 months	0.58 (1.02)		0.47 (0.85)		0.69 (1.14)	
	6 months	0.56 (1.01)		0.45 (0.88)		0.65 (1.10)	
	12 months	0.59 (1.07)		0.57 (0.99)		0.62 (1.14)	
**Number of days with 5 or more drinks in a typical drinking week, median (IQR)**							
	Baseline	0 (0-2.00)		0 (0-1.00)		0 (0-2.00)	
	1 month	0 (0-1.00)		0 (0-1.00)		0 (0-1.00)	
	3 months	0 (0-1.00)		0 (0-1.00)		0 (0-1.00)	
	6 months	0 (0-1.00)		0 (0-1.00)		0 (0-1.00)	
	12 months	0 (0-1.00)		0 (0-1.00)		0 (0-1.00)	
**Number of days with 5 or more drinks in a peak drinking week, mean (SD)**							
	Baseline	1.53 (1.78)		1.32 (1.62)		1.74 (1.90)	
	1 month	1.02 (1.33)		1.01 (1.44)		1.03 (1.23)	
	3 months	1.04 (1.38)		0.95 (1.28)		1.11 (1.47)	
	6 months	1.06 (1.46)		0.98 (1.29)		1.13 (1.59)	
	12 months	1.23 (1.54)		1.18 (1.44)		1.27 (1.64)	
**Number of days with 5 or more drinks in a peak drinking week, median (IQR)**							
	Baseline	1.00 (0-2.00)		1.00 (0-2.00)		1.00 (0-3.00)	
	1 month	1.00 (0-2.00)		0.00 (0-1.00)		1.00 (0-2.00)	
	3 months	0.50 (0-2.00)		1.00 (0-2.00)		0 (0-2.00)	
	6 months	1.00 (0-2.00)		1.00 (0-1.00)		0.50 (0-2.00)	
	12 months	1.00 (0-2.00)		1.00 (0-2.00)		1.00 (0-2.00)	
**Number of any days with 5 or more drinks in 1 event in the past month, n (%)**							
	Baseline	193 (58.7)		85 (52.5)		108 (64.7)	
	1 month	141 (51.1)		65 (49.2)		76 (52.8)	
	3 months	131 (48.5)		62 (48.1)		69 (48.9)	
	6 months	128 (49.8)		60 (49.6)		68 (50.0)	
	12 months	125 (53.4)		61 (53.5)		64 (53.3)	

^a^eSBI: electronic screening and brief intervention.

### Primary Outcomes

**Figure 3:**
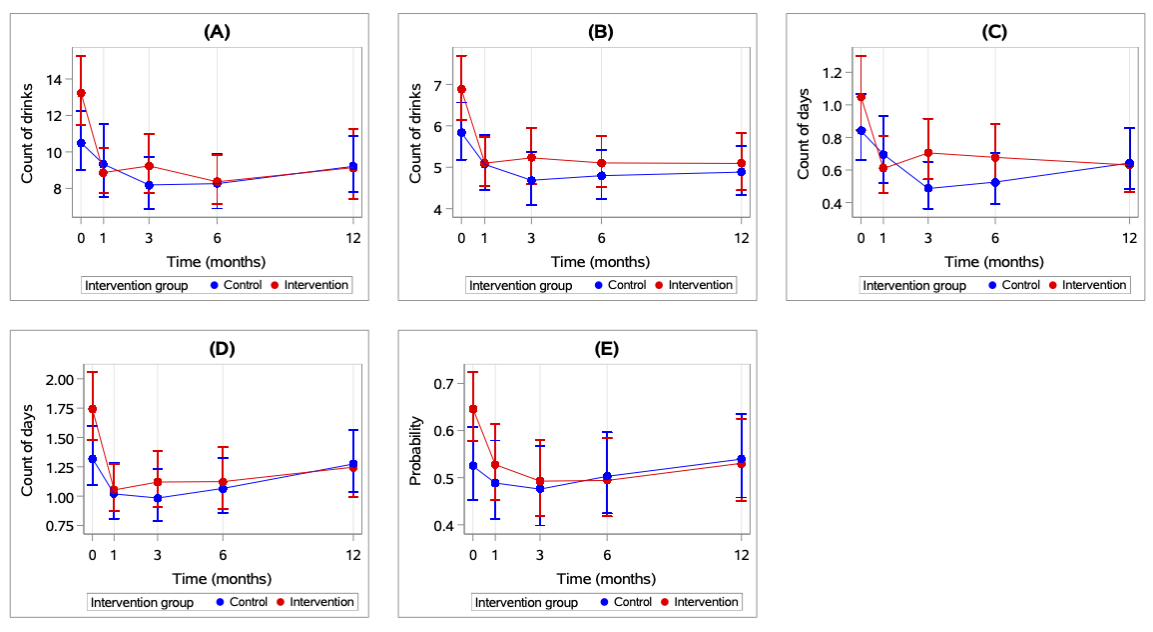
Primary outcome at each time point (0, 1, 3, 6, and 12 months) by intervention group based on generalized estimating equation (GEE) models: (A) number of drinks in a typical drinking week in the past 30 days, (B) number of drinks in a peak drinking week in the past 30 days, (C) number of days with 5 or more drinks in a typical drinking week in the past 30 days, (D) number of days with 5 or more drinks in a peak drinking week in the past 30 days, (E) any episodes with 5 or more drinks in the past 30 days.

The results of the adjusted GEE models for primary outcomes are presented in Table 3. The omnibus tests (Type III Score tests) showed that the overall effect of the intervention on substance use was not statistically significant (ie, no significant differences between groups across all time points) for any of the primary outcomes. The overall effect of time was statistically significant only for binge drinking in a peak drinking week at 12 months compared with 1 month (ie, there was an increase in drinking outcomes over time across both groups; IRR=1.32, 95% CI 1.05-1.66), and the intervention group-by-time interaction was not statistically significant (ie, differences between groups did not vary over time) for any of the primary outcomes. Figure 3 displays the results of the GEE models with the predicted values and lower and upper 95% CIs.

**Table 3:** Primary outcomes from the generalized estimating equation estimates of the treatment effect.

Model^a^				Incidence rate ratio (95% CI)		*P* value^b^
**Number of drinks in a typical drinking week**						
	**Treatment group**					.49^c^
		eSBI^d^	0.97 (0.80-1.16)		.71	
	**Time**					.56^c^
		1 month	Reference		Reference	
		3 months	0.98 (0.83-1.14)		.74	
		6 months	0.98 (0.85-1.14)		.80	
		12 months	1.12 (0.97-1.30)		.14	
	**Treatment effects (group × time)^e^**					.55^c^
		3 months to 1 month	1.04 (0.84-1.27)		.72	
		6 months to 1 month	1.00 (0.81-1.24)		.97	
		12 months to 1 month	0.89 (0.72-1.10)		.29	
**Number of drinks in a peak drinking week**						
	**Treatment group**					.88^c^
		eSBI	0.98 (0.84-1.14)		.78	
	**Time**					.98^c^
		1 month	Reference		Reference	
		3 months	0.95 (0.83-1.10)		.50	
		6 months	0.96 (0.86-1.06)		.43	
		12 months	1.00 (0.89-1.12)		.96	
	**Treatment effects (group × time)^e^**					.82^c^
		3 months to 1 month	1.06 (0.88-1.28)		.52	
		6 months to 1 month	1.06 (0.91-1.24)		.43	
		12 months to 1 month	1.00 (0.84-1.19)		.99	
**Number of days with 5 or more drinks in a typical drinking week**						
	**Treatment group**					.76^c^
		eSBI	0.88 (0.60-1.28)		.49	
	**Time**					.85^c^
		1 month	Reference		Reference	
		3 months	0.75 (0.54-1.05)		.10	
		6 months	0.82 (0.58-1.15)		.24	
		12 months	1.02 (0.73-1.41)		.92	
	**Treatment effects (group × time)^e^**					.13^c^
		3 months to 1 month	1.52 (0.99-2.31)		.056	
		6 months to 1 month	1.36 (0.88-2.13)		.17	
		12 months to 1 month	0.98 (0.63-1.53)		.94	
**Number of days with 5 or more drinks in a peak drinking week**						
	**Treatment group**					.62^c^
		eSBI	0.98 (0.75-1.28)		.87	
	**Time**					.047^c^
		1 month	Reference		Reference	
		3 months	1.02 (0.83-1.26)		.84	
		6 months	1.13 (0.89-1.43)		.33	
		12 months	1.32 (1.05-1.66)		.02	
	**Treatment effects (group × time)^e^**					.94^c^
		3 months to 1 month	1.00 (0.76-1.33)		.97	
		6 months to 1 month	0.95 (0.69-1.31)		.77	
		12 months to 1 month	0.92 (0.66-1.27)		.60	
**Number of any days with 5 or more drinks in 1 event in the past month**						
	**Treatment group**					.33^c^
		eSBI	0.99 (0.81-1.21)		.92	
	**Time**					.72^c^
		1 month	Reference		Reference	
		3 months	0.97 (0.80-1.18)		.77	
		6 months	1.02 (0.86-1.22)		.80	
		12 months	1.08 (0.92-1.26)		.37	
	**Treatment effects (group × time)^e^**					.75^c^
		3 months to 1 month	0.97 (0.76-1.23)		.79	
		6 months to 1 month	0.89 (0.69-1.15)		.37	
		12 months to 1 month	0.90 (0.71-1.15)		.40	

^a^Each model also controlled for the baseline value of the outcome variable to control for baseline imbalances between the groups.

^b^Omnibus test *P* values were from Score tests; all other *P* values were from Wald tests.

^c^Omnibus *P* values.

^d^eSBI: electronic screening and brief intervention.

^e^The ratio of the intervention to control at the indicated time point versus baseline.

### Secondary Outcomes

**Figure 4:**
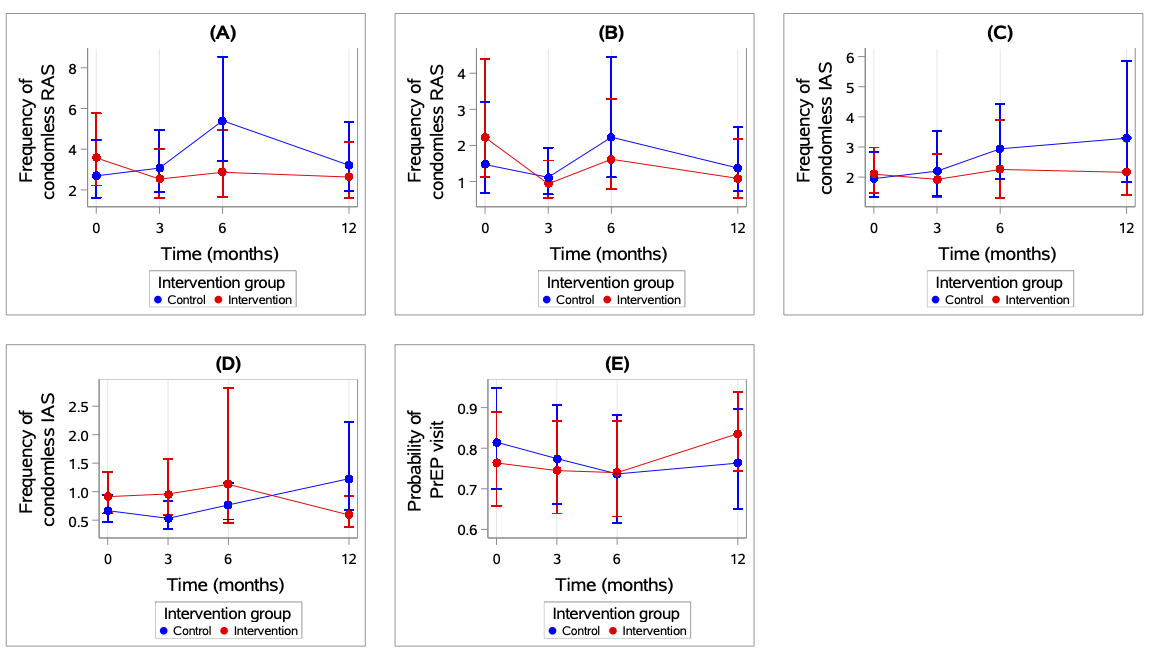
Secondary outcome at each time point (0, 3, 6, and 12 months) by intervention group based on generalized estimating equation (GEE) models: (A) number of times of condomless receptive anal sex (RAS), (B) number of times of condomless RAS while under the influence of alcohol or drugs, (C) number of times of condomless insertive anal sex (IAS), (D) number of times of condomless IAS while under the influence of alcohol or drugs, (E) any visits to a provider for pre-exposure prophylaxis (PrEP).

The results of the adjusted GEE models for secondary outcomes are presented in Table 4. The intervention group showed a reduction in the number of condomless IAS acts while under the influence of alcohol or drugs, whereas the control group showed an increase in the number of acts from 3 months to 12 months (IRR=0.15, 95% CI 0.05-0.44) when examining the contrasts. There were no significant differences between the groups in the other sexual behavior outcomes or number of PrEP care visits between groups. The results of the GEE models are visualized in Figure 4.

In terms of acceptability and satisfaction, 89.7% (148/167) of intervention participants rated the quality of the intervention as “good” to “excellent,” and 94.6% (156/167) indicated they were “mostly satisfied” to “very satisfied.” A total of 89.7% (148/167) indicated that they would “probably” or “definitely” refer a friend to receive the intervention. The most common suggestions for improvement to the intervention were to include interaction with an educator or counselor (n=8); make the intervention less prescriptive for low levels of drinking (n=10); and include graphics, video, or music (n=3).

**Table 4:** Secondary outcomes from the generalized estimating equation estimates of the treatment effect.

Model^a^					Incidence rate ratio (95% CI)		*P* value^b^
**Number of times of condomless receptive anal sex in the past 3 monthss**							
		**Treatment group**					.24^c^
			eSBI	0.79 (0.46-1.36)		.40	
		**Time**					.16^c^
			3 months	Reference		Reference	
			6 months	1.76 (1.26-2.45)		.001	
			12 months	1.17 (0.75-1.83)		.49	
		**Treatment effects (group × time)^d^**					.26^c^
			6 months to 3 months	0.66 (0.37-1.17)		.16	
			12 months to 3 months	1.03 (0.51-2.07)		.94	
**Number of times of condomless receptive anal sex while under the influence of alcohol or drugs**							
		**Treatment group**					.33^c^
			eSBI	0.80 (0.45-1.41)		.43	
		**Time**					.20^c^
			3 months	Reference		Reference	
			6 months	1.87 (1.14-3.05)		.013	
			12 months	1.33 (0.81-2.17)		.26	
		**Treatment effects (group × time)^d^**					.49^c^
			6 months to 3 months	0.74 (0.32-1.71)		.48	
			12 months to 3 months	1.10 (0.42-2.91)		.84	
**Number of times of condomless insertive anal sex in the past 3 months**							
		**Treatment group**					.29^c^
			eSBI	1.08 (0.61-1.89)		.79	
		**Time**					.19^c^
			3 months	Reference		Reference	
			6 months	1.48 (1.06-2.05)		.02	
			12 months	1.90 (1.11-3.26)		.02	
		**Treatment effects (group × time)^d^**					.25^c^
			6 months to 3 months	0.73 (0.32-1.65)		.44	
			12 months to 3 months	0.53 (0.25-1.12)		.10	
**Number of times of condomless insertive anal sex while under influence of alcohol or drugs**							
		**Treatment group**					.94^c^
			eSBI	2.48 (1.14-5.39)		.02	
		**Time**					.32^c^
			3 months	Reference		Reference	
			6 months	2.39 (1.45-3.95)		.001	
			12 months	3.58 (1.69-7.56)		.001	
		**Treatment effects (group × time)^d^**					.06^c^
			6 months to 3 months	0.41 (0.12-1.38)		.15	
			12 months to 3 months	0.15 (0.05-0.44)		.001	
**At least 1 PrEP^e^** **care visit in a 3-month period^f^**							
	**Treatment group**						.09^c^
			eSBI	1.05 (0.80-1.37)		0.728	
	**Time**						.25^c^
			3 months	Reference		Reference	
			6 months	0.83 (0.63-1.10)		.19	
			12 months	0.99 (0.74-1.32)		.94	
	**Treatment effects (group × time)^d^**						.41^c^
			6 months to 3 months	1.28 (0.89-1.84)		.18	
			12 months to 3 months	1.17 (0.84-1.64)		.36	

^a^Each model also controlled for the baseline value of the outcome variable to control for baseline imbalances between the groups.

^b^Omnibus test *P* values were from Score tests; all other *P* values were from Wald tests.

^c^Omnibus *P* values.

^d^The ratio of the intervention to control at the indicated time point versus baseline.

^e^PrEP: pre-exposure prophylaxis.

^f^Only including time points after the first indicated PrEP use.

### Moderation Effects

As an exploratory aim, we conducted tests of moderation to determine whether the efficacy of the intervention was modified by the presence of depression or anxiety. The observed effects for anxiety were unstable and varied in direction, magnitude, and statistical significance depending on the analytic approach. When we controlled for baseline imbalances in the outcomes, the intervention appeared to be more efficacious among participants with fewer symptoms of anxiety; however, when we did not control for baseline imbalances, the intervention appeared more efficacious among participants with more anxiety symptoms. The contradictory findings may be due to associations between anxiety and the baseline outcomes, but the result is difficult to interpret and should be considered with caution. No moderation effects were observed for depression in any of the analyses.

## Discussion

### Principal Findings

In this study, we found no effect of the eSBI intervention on alcohol misuse, but there was evidence of an effect on sexual risk. This study was born from a desire to create a low-cost, scalable, and brief single time-point intervention to address substance misuse in community settings. Prior literature has shown that computerized interventions can successfully reduce substance misuse using an MI approach. For example, Ondersma and colleagues [20] demonstrated that a single 20-minute eSBI intervention, with a more comprehensive MI approach, successfully improved abstinence from drugs at 3 months and 6 months among postpartum drug users. In contrast, our eSBI approach failed to show any major effect of eSBI on drinking behavior in moderate to high-risk drinkers. Although there was an effect at 12 months with significant increase in the number of binge drinking episodes in a peak week for both the eSBI group and our control condition, these differences were not seen at 3 months or 6 months, and the time-by-group effect was not significant. Our intervention’s lack of effect, in contrast to the results by Ondersma et al [20], may be the result of differing populations (HIV at-risk YMSM and YTW vs postpartum women) and the need for cultural tailoring of the intervention for the former. In addition, the trial by Ondersma et al [20] used animation to deliver the intervention, which may have improved engagement with the content.

Since this study was conceived, there has been a number of trials of eSBI that have found no effect [21,22] or some impact in particular populations including college age drinkers [23], nonpregnant women of childbearing age [24], individuals on antiretroviral therapy [25], and graduate students using cannabis [26]. The literature on eSBI can best be described as mixed, and there is evidence that the more efficacious eSBI interventions have strong cultural tailoring, which our intervention did not have due to a desire to scale this intervention broadly. In addition, our MI approach was very brief and included relatively short MI components. It was by design simple, and we were seeking to test a system that might be broadly implemented. However, this brevity may have come with a price, as the intervention may not have had the robustness of a full MI intervention.

Despite the lack of findings for substance misuse, there was evidence of reduction in sexual risk in the intervention group in comparison to the control group for condomless IAS under the influence at 12 months. These analyses should be interpreted with caution, as there were multiple secondary analyses. Nevertheless, this finding is intriguing and should be the subject of additional study.

At the time that this study was conceived in 2014, trials had mixed findings on the impact of brief interventions on AOD. Since that time, observers have raised criticism of the value of screening and brief interventions [27-29]. The Substance Abuse and Mental Health Services Administration (SAMHSA)–promoted SBIRT model has generally shown impact on individuals with high levels of substance use and in primary care settings [27]. It is possible that our intervention was too brief and lacking in boosters or follow-up. Further trials may be merited with these elements in a dynamic trial design that might allow simultaneous comparisons.

Another promising line of inquiry that has shown impact is the use of relational agents (ie, using avatars, conversational bots, or other interfaces) that simulate face-to-face dynamics and allow for a more holistic experience during screening and a brief intervention [30-32]. Relative to this type of relational agent, our intervention was static; designed with simple, scalable elements; and presented in a slide format. The simplistic design was by intention, as we hypothesized that many clinics would not have the infrastructure, computing capabilities, and high bandwidth access that are often required for more computationally complex frameworks. We suspect that our eSBI system lacked enough appealing components to produce a robust attentional response; however, as we did not measure attention to the eSBI system, this is purely speculative.

Although, overall, the intervention received high ratings for acceptability and satisfaction, some participants suggested the addition of more dynamic and engaging components (eg, more graphics, video, and music), commented that the fully automated version was impersonal and would have preferred more interaction with an educator or counselor, or felt the intervention was too prescriptive for perceived low levels of drinking. These are important considerations to inform future iterations in this population.

Finally, since we conceptualized this study in 2014, eSBI approaches have been tested for feasibility among people living with HIV in sub-Saharan Africa, where alcohol misuse is associated with both HIV transmission risk as well as poor HIV treatment outcomes but is often underreported [25,33]. In Uganda and Namibia, an electronic screening approach was designed specifically to increase reporting of alcohol misuse and, in Namibia, was then coupled with brief intervention. In both studies, evidence of feasibility was apparent, although with concerns about confidentiality (ie, providers being aware of drinking levels), which hampered acceptability [25,33]. These types of initiatives will provide important additional tests of effectiveness of eSBI to reduce alcohol misuse in HIV care environments and, given our findings, may also hold promise for reducing the risk for ongoing HIV transmission (ie, via sexual risk reduction).

### Conclusions

This study found a lack of longer-term impact of a single time point intervention, eSBI, on alcohol misuse among YMSM and YTW presenting to community-based HIV testing clinics. The intervention showed an effect in reducing one of the sexual risk outcomes (number of condomless IAS acts while under the influence). Given that this intervention was embedded within larger systems of care (ie, placed within HIV testing clinics without the need for staffing), there would appear to be some value in considering further trials in this space. Further adaptation of the eSBI approach might result in greater impact. This eSBI intervention was embedded within an STTR model of care that has been shown to be highly effective in addressing HIV risk and engaging individuals in care. The study showed excellent recruitment and retention of the sample over a 1-year follow-up period. Our eSBI system can be considered a branch of the broader SBIRT model of care. This model has come under some criticism in recent years due to a lack of compelling evidence to show its impact as a primary prevention and intervention approach. Our eSBI system may have failed to achieve the intended goals of alcohol misuse reduction because of the brevity of the intervention, the lack of boosters or follow-up, and the relatively simple design of the intervention. Regardless of the reason, this study falls into alignment with other negative trials of SBIRT. Future research may need to consider relational agents, digital interventions hybridized with human counselors or therapists, and interventions with boosters of some type. In addition, interventions that can address contextual factors (interpersonal, social, and environmental) may help boost the effect of digital interventions like eSBI. Given the instability of results for moderation of the intervention effect by symptoms of anxiety, future studies may need larger samples or be limited to the subpopulation of individuals with higher levels of anxiety to explore these effects more fully.

## Appendix

Multimedia Appendix 1 Table S1: generalized estimating equation (GEE) estimates of treatment effect unadjusted for baseline difference in alcohol use.

Multimedia Appendix 2 CONSORT-eHEALTH checklist (V 1.6.2).

## Abbreviations

AOD: alcohol and other drugs
AUDIT: Alcohol Use Disorders Identification Test
CASI: computer-assisted self-interviewing
CES-D10: Center for Epidemiologic Studies Depression Scale 10
CSQ-8: Client Satisfaction Questionnaire
DDQ-R: Daily Drinking Questionnaire Revised
eSBI: electronic screening and brief intervention
GAD-7: Generalized Anxiety Disorder 7
GEE: generalized estimating equation
IAS: insertive anal sex
IRR: incidence rate ratio
MI: motivational interviewing
PrEP: pre-exposure prophylaxis
RAS: receptive anal sex
RR: relative risk
SAMHSA: Substance Abuse and Mental Health Services Administration
SBIRT: screening, brief intervention, and referral to treatment
STI: sexually transmitted infection
STTR: seek, test, treat, and retain
YMSM: young men who have sex with men
YTW: young transgender women
