# Supplementary material for: Findings From the Step Up, Test Up Study of an Electronic Screening and Brief Intervention for Alcohol Misuse in Adolescents and Young Adults Presenting for HIV Testing: Randomized Controlled Efficacy Trial
Source: JMIR Ment Health. 2023 Mar 29;10:e43653. doi: 10.2196/43653 (PMC10131684; doi:10.2196/43653)
Supplement: Multimedia Appendix 1 [file mental_v10i1e43653_app1.docx]

Supplementary Table 1: GEE Estimates of Treatment Effect Unadjusted for Baseline Difference in Alcohol Use

|  | *IRR* | *95% CI* | *p* |
| --- | --- | --- | --- |
| # Drinks in a Typical Drinking Week |  |  |  |
| *Treatment Group* |  |  | *Omnibus:* 0.539 |
| eSBI | 1.26 | 1.02 - 1.55 | 0.031 |
| *Time* |  |  | *Omnibus:* <0.001 |
| Baseline | REF | REF | REF |
| 1 Month | 0.89 | 0.76 - 1.03 | 0.123 |
| 3 Months | 0.78 | 0.68 - 0.90 | <0.001 |
| 6 Months | 0.79 | 0.67 - 0.92 | 0.003 |
| 12 Months | 0.88 | 0.75 - 1.02 | 0.085 |
| *Treatment Effects (Group × Time) ^a^* |  |  | *Omnibus:* 0.137 |
| 1 Month to Baseline | 0.76 | 0.61 - 0.93 | 0.008 |
| 3 Months to Baseline | 0.89 | 0.72 - 1.11 | 0.301 |
| 6 Months to Baseline | 0.80 | 0.63 - 1.02 | 0.076 |
| 12 Months to Baseline | 0.79 | 0.61 - 1.01 | 0.061 |
| # Drinks in a Peak Drinking Week |  |  |  |
| *Treatment Group* |  |  | *Omnibus:* 0.260 |
| eSBI | 1.18 | 1.00 - 1.39 | 0.049 |
| *Time* |  |  | *Omnibus:* <0.001 |
| Baseline | REF | REF | REF |
| 1 Month | 0.87 | 0.77 - 0.98 | 0.020 |
| 3 Months | 0.80 | 0.70 - 0.93 | 0.003 |
| 6 Months | 0.82 | 0.73 - 0.92 | 0.001 |
| 12 Months | 0.84 | 0.74 - 0.95 | 0.006 |
| *Treatment Effects (Group × Time) ^a^* |  |  | *Omnibus:* 0.493 |
|  | *IRR* | *95% CI* | *p* |
| 1 Month to Baseline | 0.85 | 0.72 - 1.02 | 0.075 |
| 3 Months to Baseline | 0.95 | 0.78 - 1.15 | 0.585 |
| 6 Months to Baseline | 0.90 | 0.75 - 1.08 | 0.274 |
| 12 Months to Baseline | 0.88 | 0.73 - 1.07 | 0.217 |
| # Days 5+ Drinks in a Typical Drinking Week |  |  |  |
| *Treatment Group* |  |  | *Omnibus:* 0.362 |
| eSBI | 1.25 | 0.90 - 1.72 | 0.179 |
| *Time* |  |  | *Omnibus:* <0.001 |
| Baseline | REF | REF | REF |
| 1 Month | 0.83 | 0.65 - 1.06 | 0.134 |
| 3 Months | 0.58 | 0.44 - 0.77 | <0.001 |
| 6 Months | 0.63 | 0.48 - 0.83 | 0.001 |
| 12 Months | 0.77 | 0.58 - 1.01 | 0.059 |
| *Treatment Effects (Group × Time) ^a^* |  |  | *Omnibus:* 0.076 |
| 1 Month to Baseline | 0.70 | 0.48 - 1.02 | 0.062 |
| 3 Months to Baseline | 1.16 | 0.80 - 1.69 | 0.437 |
| 6 Months to Baseline | 1.03 | 0.70 - 1.52 | 0.872 |
| 12 Months to Baseline | 0.79 | 0.53 - 1.18 | 0.242 |
| # Days 5+ Drinks in a Peak Drinking Week |  |  |  |
| *Treatment Group* |  |  | *Omnibus:* 0.416 |
| eSBI | 1.32 | 1.03 - 1.69 | 0.030 |
| *Time* |  |  | *Omnibus:* <0.001 |
| Baseline | REF | REF | REF |
| 1 Month | 0.77 | 0.65 - 0.91 | 0.002 |
| 3 Months | 0.74 | 0.61 - 0.91 | 0.004 |
|  | *IRR* | *95% CI* | *p* |
| 6 Months | 0.81 | 0.66 - 0.99 | 0.040 |
| 12 Months | 0.96 | 0.81 - 1.15 | 0.690 |
| *Treatment Effects (Group × Time) ^a^* |  |  | *Omnibus:* 0.260 |
| 1 Month to Baseline | 0.78 | 0.60 - 1.01 | 0.064 |
| 3 Months to Baseline | 0.86 | 0.65 - 1.14 | 0.300 |
| 6 Months to Baseline | 0.80 | 0.58 - 1.09 | 0.162 |
| 12 Months to Baseline | 0.74 | 0.55 - 1.00 | 0.050 |
|  | *RR* | *95% CI* | *p* |
| Any Days 5+ Drinks in one event past month |  |  |  |
| *Treatment Group* |  |  | *Omnibus:* 0.482 |
| eSBI | 1.23 | 1.02 - 1.48 | 0.027 |
| *Time* |  |  | *Omnibus:* 0.028 |
| Baseline | REF | REF | REF |
| 1 Month | 0.93 | 0.78 - 1.11 | 0.429 |
| 3 Months | 0.91 | 0.75 - 1.09 | 0.297 |
| 6 Months | 0.96 | 0.80 - 1.15 | 0.645 |
| 12 Months | 1.03 | 0.86 - 1.23 | 0.765 |
| *Treatment Effects (Group × Time) ^a^* |  |  | *Omnibus:* 0.355 |
| 1 Month to Baseline | 0.88 | 0.69 - 1.11 | 0.265 |
| 3 Months to Baseline | 0.84 | 0.66 - 1.07 | 0.161 |
| 6 Months to Baseline | 0.80 | 0.62 - 1.03 | 0.078 |
| 12 Months to Baseline | 0.80 | 0.62 - 1.03 | 0.079 |
| Secondary Outcomes |  |  |  |
|  | *IRR* | *95% CI* | *p* |
| # Times Condomless RAS in past 3 Months |  |  |  |
| *Treatment Group* |  |  | *Omnibus: 0.522* |
| eSBI | 1.33 | 0.66 - 2.66 | *0.424* |
| *Time* |  |  | *Omnibus:0.246* |
| Baseline | REF | REF | REF |
| 3 Months | 1.14 | 0.69 – 1.90 | 0.609 |
| 6 Months | 2.01 | 1.37 – 2.93 | < 0.001 |
| 12 Months | 1.19 | 0.68 – 2.08 | 0.535 |
| *Treatment Effects (Group × Time) ^a^* |  |  | *Omnibus:0.030* |
| 3 Months to Baseline | 0.62 | 0.31 – 1.25 | 0.184 |
| 6 Months to Baseline | 0.40 | 0.22 – 0.72 | 0.002 |
| 12 Months to Baseline | 0.62 | 0.27 – 1.40 | 0.250 |
| # Times Condomless RAS While Under Influence of Alcohol and/or Drugs |  |  |  |
| *Treatment Group* |  |  | *Omnibus: 0.847* |
| eSBI | 1.51 | 0.54 – 4.25 | 0.433 |
| *Time* |  |  | *Omnibus: 0.250* |
| Baseline | REF | REF | REF |
| 3 Months | 0.76 | 0.54 – 4.25 | 0.389 |
| 6 Months | 1.52 | 1.07 – 2.15 | 0.020 |
| 12 Months | 0.93 | 0.49 – 1.79 | 0.837 |
| *Treatment Effects (Group × Time) ^a^* |  |  | *Omnibus: 0.076* |
| 3 Months to Baseline | 0.55 | 0.22 – 1.42 | 0.217 |
| 6 Months to Baseline | 0.48 | 0.28 – 0.81 | 0.006 |
| 12 Months to Baseline | 0.52 | 0.17 – 1.59 | 0.254 |
| # Times Condomless IAS in past 3 Months |  |  |  |
| *Treatment Group* |  |  | *Omnibus: 0.418* |
| eSBI | 1.08 | 0.65 – 1.79 | 0.777 |
| *Time* |  |  | *Omnibus: 0.250* |
| Baseline | REF | REF | REF |
| 3 Months | 1.13 | 0.72 – 1.76 | 0.598 |
| 6 Months | 1.51 | 1.04 – 2.19 | 0.031 |
| 12 Months | 1.69 | 0.91 – 3.12 | 0.095 |
| *Treatment Effects (Group × Time)* |  |  | *Omnibus: 0.473* |
| 3 Months to Baseline | 0.81 | 0.45 – 1.47 | 0.494 |
| 6 Months to Baseline | 0.71 | 0.38 – 1.35 | 0.296 |
| 12 Months to Baseline | 0.61 | 0.30 – 1.26 | 0.179 |
| # Times Condomless IAS While Under Influence of Alcohol and/or Drugs |  |  |  |
| *Treatment Group* |  |  | *Omnibus:0.572* |
| eSBI | 1.37 | 0.81 – 2.30 | 0.236 |
| *Time* |  |  | *Omnibus: 0.801* |
| Baseline | REF | REF | REF |
| 3 Months | 0.80 | 0.56 – 1.13 | 0.204 |
| 6 Months | 1.15 | 0.72 – 1.84 | 0.555 |
| 12 Months | 1.84 | 0.99 – 3.42 | 0.053 |
| *Treatment Effects (Group × Time) ^a^* |  |  | *Omnibus: 0.134* |
| 3 Months to Baseline | 1.32 | 0.70 – 2.49 | 0.393 |
| 6 Months to Baseline | 1.07 | 0.38 – 3.07 | 0.894 |
| 12 Months to Baseline | 0.35 | 0.17 – 0.75 | 0.007 |
|  | *RR* | *95% CI* | *p* |
| At least 1 PrEP Care Visit in 3 Mo. Period ^b^ |  |  |  |
| *Treatment Group* |  |  | *Omnibus: 0.976* |
| eSBI | 0.94 | 0.76 – 1.16 | 0.555 |
| *Time* |  |  | *Omnibus: 0.697* |
| Baseline | REF | REF | REF |
| 3 Months | 0.95 | 0.78 – 1.16 | 0.623 |
| 6 Months | 0.90 | 0.71 – 1.15 | 0.403 |
| 12 Months | 0.94 | 0.75 – 1.18 | 0.573 |
| *Treatment Effects (Group × Time) ^a^* |  |  | *Omnibus: 0.744* |
| 3 Months to Baseline | 1.03 | 0.77 – 1.37 | 0.860 |
| 6 Months to Baseline | 1.07 | 0.79 – 1.45 | 0.658 |
| 12 Months to Baseline | 1.17 | 0.88 – 1.56 | 0.289 |

*Note:* Omnibus test p-values from Score tests; all other p-values from Wald tests.

a The ratio of the ratio of the intervention to. control at the indicated time point vs. baseline.

b Only including time points after first indicated PrEP use.
