# Supplementary material for: Findings From the Step Up, Test Up Study of an Electronic Screening and Brief Intervention for Alcohol Misuse in Adolescents and Young Adults Presenting for HIV Testing: Randomized Controlled Efficacy Trial
Source: JMIR Ment Health. 2023 Mar 29;10:e43653. doi: 10.2196/43653 (PMC10131684; doi:10.2196/43653)
Supplement: Multimedia Appendix 2 [file mental_v10i1e43653_app2.pdf]

CONSORT-EHEALTH Checklist V1.6.2 Report	Manuscript Number	43653
**(based on CONSORT-EHEALTH V1.6), available at [http://tinyurl.com/consort-ehealth-v1-6].**		
**Date completed**		
2/15/2023 18:36:46		
**by**		
Kuhns		
Findings from the step-up, test up study of an electronic screening and brief intervention for alcohol misuse in adolescents and young adults presenting for HIV testing: A randomized controlled efficacy trial		
TITLE		
**1a-i) Identify the mode of delivery in the title**		
"Findings from the Step Up, Test Up Study of an electronic screening and brief intervention for alcohol misuse in adolescents and young adults presenting for HIV testing: A randomized controlled efficacy trial "		
**1a-ii) Non-web-based components or important co-interventions in title**		
This is not relevant to this manuscript		
**1a-iii) Primary condition or target group in the title**		
"Findings from the Step Up, Test Up Study of an electronic screening and brief intervention for alcohol misuse in adolescents and young adults presenting for HIV testing: A randomized controlled efficacy trial "		
ABSTRACT		
**1b-i) Key features/functionalities/components of the intervention and comparator in the METHODS section of the ABSTRACT**		
"Youth ages 16-25 who presented for HIV testing at community-based locations were recruited for study participation. Those who screened at moderate to high risk on the Alcohol Use Disorders Identification Test were randomized (1:1) to either an electronic brief intervention or a time-attention control. "		
**1b-ii) Level of human involvement in the METHODS section of the ABSTRACT**		
"The purpose of the study was to test the efficacy of a fully automated electronic screening and brief intervention, called Step Up, Test Up, to reduce alcohol misuse among adolescents and young adults presenting for HIV testing. Secondary objectives were reduction in sexual risk and uptake of pre-exposure prophylaxis (PrEP) for HIV prevention."		
**1b-iii) Open vs. closed, web-based (self-assessment) vs. face-to-face assessments in the METHODS section of the ABSTRACT**		
"Youth ages 16-25 who presented for HIV testing at community-based locations were recruited for study participation. Those who screened at moderate to high risk on the Alcohol Use Disorders Identification Test were randomized (1:1) to either an electronic brief intervention or a time-attention control. The primary outcome was change in alcohol use at 1, 3, 6 and 12-month follow-up. Negative binomial and log binomial regression with generalized estimating equations were conducted to evaluate the intervention efficacy."		
**1b-iv) RESULTS section in abstract must contain use data**		
"Among a sample of 329 youth, there were no significant differences in alcohol use outcomes between conditions over time or at 1, 3, 6 or 12-month timepoints. In terms of secondary outcomes, there was evidence of reduction in condomless insertive anal sex under the influence of alcohol and drugs at 12 months compared to 3 months in the intervention versus the attention control condition (IRR 0.15, 95% CI 0.05-0.44), however there were no other significant differences in sexual risk and no difference in PrEP engagement. "		
**1b-v) CONCLUSIONS/DISCUSSION in abstract for negative trials**		
"We found no effect of electronic brief intervention to reduce alcohol use, and some effect on sexual risk among youth ages 16-25 who present for HIV testing. "		
INTRODUCTION		
**2a-i) Problem and the type of system/solution**		
"The purpose of this study was to assess the feasibility, acceptability, and initial efficacy of eSBI in comparison to an electronic attention control intervention (i.e., promotion of good nutrition), coupled with standard HIV prevention, on alcohol use among YMSM and YTW in community-based HIV testing environments in Chicago. Secondary objectives were to assess intervention effects on sexual behavior, as well as engagement within the HIV prevention and care continuum, and to assess modification of the intervention effect by co-morbid mental health problems (i.e., symptoms of depression, and anxiety)."		
**2a-ii) Scientific background, rationale: What is known about the (type of) system**		
"At the intersection of individual behavioral change and structural change are attempts to insert brief motivational strategies into larger systems of care. Motivational interviewing (MI)-based approaches promote screening, brief intervention, and referral to treatment (aka SBIRT). Electronic screening and brief intervention (eSBI) is a subset of SBIRT in an electronic medium suitable for use in primary care and other generalist settings. YMSM and YTW often seek HIV testing and other supportive services in community-based and outreach settings. These settings are underutilized as potential entry points for engagement in comprehensive care across the HIV prevention and care continuum, including pre-exposure prophylaxis (PrEP) for HIV-negative youth who are at risk of HIV acquisition. We theorized that these same community settings could serve as an access point for substance use interventions if these interventions were brief and scalable."		
**Does your paper address CONSORT subitem 2b?**		
"The purpose of this study was to assess the feasibility, acceptability, and initial efficacy of eSBI in comparison to an electronic attention control intervention (i.e., promotion of good nutrition), coupled with standard HIV prevention, on alcohol use among YMSM and YTW in community-based HIV testing environments in Chicago. Secondary objectives were to assess intervention effects on sexual behavior, as well as engagement within the HIV prevention and care continuum, and to assess modification of the intervention effect by co-morbid mental health problems (i.e., symptoms of depression, and anxiety)."		
METHODS		
**3a) CONSORT: Description of trial design (such as parallel, factorial) including allocation ratio**		
"This study was a randomized controlled trial of eSBI among YMSM and YTW seeking HIV testing [8]. Using an electronic web-based portal, all participants were screened for alcohol misuse and received immediate feedback regarding their level of use (e.g., how their use compares to others, whether it exceeds “safe use” guidelines); those who screened for moderate to high alcohol use, including binge drinking, were then randomized to either electronic intervention or control modules. All participants, regardless of randomization status, were followed for 12 months with in-person visits conducted at 1, 3, 6 and 12-month intervals. "<br><br>"Participants who screened moderate to high risk for alcohol misuse or endorsed binge drinking on the AUDIT were randomized via computerized assignment (1:1) to either brief intervention modules to reduce alcohol misuse, eSBI, or a time-and attention-matched control (i.e., promotion of good nutrition). Study staff were blind to random assignments."		
**3b) CONSORT: Important changes to methods after trial commencement (such as eligibility criteria), with reasons**		
We did not have major changes to the design after trial commencement.		
**3b-i) Bug fixes, Downtimes, Content Changes**		
We did not have major bugs or down time for this intervention trial.		
**4a) CONSORT: Eligibility criteria for participants**		
"Individuals were eligible if they were: (a) aged 16 to 25 years, (b) interested in testing for HIV infection and (c) HIV-negative or HIV status unknown (per self-report; verified at the point of HIV testing), and (d) identified as MSM or a transgender woman who has sex with men (i.e., born male, identify as female/transgender, and at any point in the gender transition process); and (e) English-speaking."		
**4a-i) Computer / Internet literacy**		
We targeted a young, English-speaking population in the US, so computer literacy was ubiquitous.		
**4a-ii) Open vs. closed, web-based vs. face-to-face assessments:**		
"Participants completed a baseline study visit which included standardized assessment via computer-assisted self-interviewing (CASI). Follow-up CASI assessments, were conducted at 1, 3, 6, and 12-month follow-ups visits either in person or remotely via web-based assessment."		
**4a-iii) Information giving during recruitment**		
"All participants were recruited from HIV testing clinics that utilized a Seek, Test, Treat, Retain (STTR) model of care [9]. The protocol was approved by the Institutional Review Boards of participating institutions with a waiver of parental permission for participation of minors (aged 16-17). All enrolled participants were consented prior to the start of any research activities. "		
**4b) CONSORT: Settings and locations where the data were collected**		
"Youth were recruited from HIV testing centers at the three primary sites in Chicago: the Division of Adolescent Medicine at Lurie Children’s Hospital, Howard Brown Health, and the Village at the University of Chicago."		
**4b-i) Report if outcomes were (self-)assessed through online questionnaires**		
"Participants completed a baseline study visit which included standardized assessment via computer-assisted self-interviewing (CASI). Follow-up CASI assessments, were conducted at 1, 3, 6, and 12-month follow-ups visits either in person or remotely via web-based assessment."		
**4b-ii) Report how institutional affiliations are displayed**		
Institutional affiliations were not displayed.		
**5) CONSORT: Describe the interventions for each group with sufficient details to allow replication, including how and when they were actually administered**		
**5-i) Mention names, credential, affiliations of the developers, sponsors, and owners**		

"The electronic intervention and control modules were delivered using a customized eSBI platform developed by Radiant Interactive Group (Laguna Niguel, CA). Radiant uses industry-standard data encryption and security to manage Protected Health Information recorded by their system (Radiant is not an author)."		
**5-ii) Describe the history/development process**		
"Those randomized to the intervention were asked to complete lessons or exercises on 11 topical areas focused on alcohol use (e.g.: importance of change, downsides of drinking, barriers to change, strategies for cutting back, change motivators) each with a single webpage, in a motivational interviewing (MI) format. The intervention is not a full MI intervention as it was relatively simple in its presentation, but it did have components of the Elicit-Provide-Elicit approach. The intervention had been widely used in Employee Assistance Programs. The intervention content was adapted for late adolescents and young adults in terms of voice and language (e.g., open, non-judgmental, inclusive), contexts (e.g., school as well as work contexts) and reading level. "		
**5-iii) Revisions and updating**		
The intervention was not revised during the trial and only this original version was deployed.		
**5-iv) Quality assurance methods**		
The intervention is MI-based and does not include fact-based information, except for the standard feedback provided for screening on the AUDIT.		
**5-v) Ensure replicability by publishing the source code, and/or providing screenshots/screen-capture video, and/or providing flowcharts of the algorithms used**		
"The intervention was presented as a series of slides which contained an interactive element where participants could click on checkboxes, drop-down menus, and short text fields as part of the brief motivational interviewing exercises. An example slide is show in Figure 2. "		
**5-vi) Digital preservation**		
The application does not have an open and accessible URL.		
**5-vii) Access**		
"Using an electronic web-based portal, all participants were screened for alcohol misuse and received immediate feedback regarding their level of use (e.g., how their use compares to others, whether it exceeds “safe use” guidelines); those who screened for moderate to high alcohol use, including binge drinking, were then randomized to either electronic intervention or control modules. "		
**5-viii) Mode of delivery, features/functionalities/components of the intervention and comparator, and the theoretical framework**		
"Those randomized to the intervention were asked to complete lessons or exercises on 11 topical areas focused on alcohol use (e.g.: importance of change, downsides of drinking, barriers to change, strategies for cutting back, change motivators) each with a single webpage, in a motivational interviewing (MI) format. The intervention is not a full MI intervention as it was relatively simple in its presentation, but it did have components of the Elicit-Provide-Elicit approach. The intervention had been widely used in Employee Assistance Programs. The intervention content was adapted for late adolescents and young adults in terms of voice and language (e.g., open, non-judgmental, inclusive), contexts (e.g., school as well as work contexts) and reading level. The intervention was presented as a series of slides which contained an interactive element where participants could click on checkboxes, drop-down menus, and short text fields as part of the brief motivational interviewing exercises. An example slide is show in Figure 2. By design, the intervention lacked any specific cultural tailoring for YMSM or YTW other than the age-appropriate language. This was intended to facilitate rapid scaling and broad adoption across the general population if the intervention proved effective. The electronic intervention and control modules were delivered using a customized eSBI platform developed by Radiant Interactive Group (Laguna Niguel, CA). Radiant uses industry-standard data encryption and security to manage Protected Health Information recorded by their system."<br><br>"Those randomized to the control condition completed a brief time and attention-control intervention, also in MI format, of equal length (i.e., same number of intervention screens), which encouraged good nutrition but was not expected to impact alcohol use."		
**5-ix) Describe use parameters**		
This intervention is a one-time use at the point of HIV testing.		
**5-x) Clarify the level of human involvement**		
"The purpose of the study was to test the efficacy of a fully automated electronic screening and brief intervention, called Step Up, Test Up, to reduce alcohol misuse among adolescents and young adults presenting for HIV testing. Secondary objectives were reduction in sexual risk and uptake of pre-exposure prophylaxis (PrEP) for HIV prevention."		
**5-xi) Report any prompts/reminders used**		
This is not applicable as there were no prompts for follow-up.		
**5-xii) Describe any co-interventions (incl. training/support)**		
There are no co-interventions in this trial.		
**6a) CONSORT: Completely defined pre-specified primary and secondary outcome measures, including how and when they were assessed**		
"Participants completed a baseline study visit which included standardized assessment via computer-assisted self-interviewing (CASI). Follow-up CASI assessments, were conducted at 1, 3, 6, and 12-month follow-ups visits either in person or remotely via web-based assessment. Detailed contact information was collected to facilitate study retention.<br><br>Primary Outcome<br>All outcome measures were self-reported and constructed using the revised versions of the Daily Drinking Questionnaire [11], the DDQ-R [12], including questions regarding the heaviest drinking week [13]. The questionnaire asks the participant to report the number of drinks consumed for each day of the week for both a typical week and heavy drinking week in the past thirty days. The outcomes assessed for this analysis were: 1) the total number of drinks consumed in a typical drinking week, 2) the total number of drinks consumed in a peak drinking week, 3) the number of days 5 or more drinks were consumed in a typical week, 4) the number of days 5 or more drinks were consumed in a heavy drinking week, and 5) any binge drinking (i.e., 5 or more drinks consumed in one episode) in the prior month.<br><br>Secondary Outcomes<br>Sexual risk behavior was measured via a modified version of the AIDS-Risk Behavior Assessment [14] used in prior studies of YMSM and YTW [15, 16] and included:1) condomless receptive anal sex (RAS) acts, 2) condomless RAS acts while under the influence of alcohol and/or drugs, 3) condomless insertive anal sex (IAS) acts, and (4) condomless IAS acts while under the influence of alcohol and/or drugs. Condomless sex was defined as sex where “no condom was used or a condom was used but only for a part of the time”. Attending at least one provider visit for PrEP care during the observation period was additionally included as a secondary outcome for HIV-negative participants who reported using PrEP (n=89)."		
**6a-i) Online questionnaires: describe if they were validated for online use and apply CHERRIES items to describe how the questionnaires were designed/deployed**		
Not applicable. The study used computer-assisted self-interviewing.		
**6a-ii) Describe whether and how “use” (including intensity of use/dosage) was defined/measured/monitored**		
"Those randomized to the intervention were asked to complete lessons or exercises on 11 topical areas focused on alcohol use (e.g.: importance of change, downsides of drinking, barriers to change, strategies for cutting back, change motivators) each with a single webpage, in a motivational interviewing (MI) format. Pages were forced response, so all participants received all content."		
**6a-iii) Describe whether, how, and when qualitative feedback from participants was obtained**		
Qualitative feedback via interviews and focus groups were not obtained.		
**6b) CONSORT: Any changes to trial outcomes after the trial commenced, with reasons**		
"Youth were recruited from HIV testing centers at the three primary sites in Chicago: the Division of Adolescent Medicine at Lurie Children’s Hospital, Howard Brown Health, and the Village at the University of Chicago."		
**7a) CONSORT: How sample size was determined**		
**7a-i) Describe whether and how expected attrition was taken into account when calculating the sample size**		
"The analysis used an intent-to-treat approach in which all randomized participants were included for analysis. Changes in drinking levels over time were examined descriptively using means with standard deviations and medians with interquartile ranges (IQRs). The targeted sample size of N=450 was estimated to yield 80% power to detect effects for our primary (alcohol use) outcome under a range of retention levels."		
**7b) CONSORT: When applicable, explanation of any interim analyses and stopping guidelines**		

"Participants completed a baseline study visit which included standardized assessment via computer-assisted self-interviewing (CASI). Follow-up CASI assessments, were conducted at 1, 3, 6, and 12-month follow-ups visits either in person or remotely via web-based assessment. Detailed contact information was collected to facilitate study retention.<br><br>Primary Outcome<br>All outcome measures were self-reported and constructed using the revised versions of the Daily Drinking Questionnaire [11], the DDQ-R [12], including questions regarding the heaviest drinking week [13]. The questionnaire asks the participant to report the number of drinks consumed for each day of the week for both a typical week and heavy drinking week in the past thirty days. The outcomes assessed for this analysis were: 1) the total number of drinks consumed in a typical drinking week, 2) the total number of drinks consumed in a peak drinking week, 3) the number of days 5 or more drinks were consumed in a typical week, 4) the number of days 5 or more drinks were consumed in a heavy drinking week, and 5) any binge drinking (i.e., 5 or more drinks consumed in one episode) in the prior month.<br><br>Secondary Outcomes<br>Sexual risk behavior was measured via a modified version of the AIDS-Risk Behavior Assessment [14] used in prior studies of YMSM and YTW [15, 16] and included:1) condomless receptive anal sex (RAS) acts, 2) condomless RAS acts while under the influence of alcohol and/or drugs, 3) condomless insertive anal sex (IAS) acts, and (4) condomless IAS acts while under the influence of alcohol and/or drugs. Condomless sex was defined as sex where “no condom was used or a condom was used but only for a part of the time”. Attending at least one provider visit for PrEP care during the observation period was additionally included as a secondary outcome for HIV-negative participants who reported using PrEP (n=89)."		
**8a) CONSORT: Method used to generate the random allocation sequence**		
"Participants who screened moderate to high risk for alcohol misuse or endorsed binge drinking on the AUDIT were randomized via computerized assignment (1:1) to either brief intervention modules to reduce alcohol misuse, eSBI, or a time-and attention-matched control (i.e., promotion of good nutrition). Study staff were blind to random assignments."		
**8b) CONSORT: Type of randomisation; details of any restriction (such as blocking and block size)**		
Not relevant, no blocking.		
**9) CONSORT: Mechanism used to implement the random allocation sequence (such as sequentially numbered containers), describing any steps taken to conceal the sequence until interventions were assigned**		
"Participants who screened moderate to high risk for alcohol misuse or endorsed binge drinking on the AUDIT were randomized via computerized assignment (1:1) to either brief intervention modules to reduce alcohol misuse, eSBI, or a time-and attention-matched control (i.e., promotion of good nutrition). Study staff were blind to random assignments. "		
**10) CONSORT: Who generated the random allocation sequence, who enrolled participants, and who assigned participants to interventions**		
"The randomization sequence was generated by NK, uploaded to the Radiant platform, and assigned automatically in order of enrollment."<br><br>"Individuals who were eligible and interested were enrolled by study staff."		
**11a) CONSORT: Blinding - If done, who was blinded after assignment to interventions (for example, participants, care providers, those assessing outcomes) and how**		
**11a-i) Specify who was blinded, and who wasn’t**		
"Study staff were blind to random assignments."		
**11a-ii) Discuss e.g., whether participants knew which intervention was the “intervention of interest” and which one was the “comparator”**		
"The consent form indicated that the purpose of the study was to learn more about whether screening and brief intervention for substance use is effective in reducing that use."		
**11b) CONSORT: If relevant, description of the similarity of interventions**		
The interventions were not similar, just equal in length.		
**12a) CONSORT: Statistical methods used to compare groups for primary and secondary outcomes**		
"To evaluate the efficacy of the intervention, generalized estimating equations (GEE) with robust standard errors were conducted. Each outcome was regressed on intervention group, time, a group by time interaction, and the baseline value of the outcome, where the statistical significance of the interaction term indicates whether the groups differ in response to the intervention over time. The baseline outcomes were included in the model due to baseline imbalances in the outcomes between the groups. Omnibus tests (Type III Score tests) were used to assess the overall effects of the intervention, time, and whether the differences between the groups varied across time points. Contrasts were performed to assess the effect of the intervention compared to the time-and-attention matched control at each follow-up visit compared to first follow-up for exploratory purposes even when Omnibus tests were not significant. Interaction contrasts represent the relative risk (RR; for binary outcomes) or rate ratio (IRR; for count outcomes) for the outcome in the intervention arm at that time point compared to the RR or IRR for the group comparison at first follow-up (i.e., a ratio of ratios). All count outcomes were modeled as negative binomially distributed variables with log link functions to compute rate ratios; the binary outcome (having had more than 5 drinks in any single drinking session in the prior month) was modeled as a binomially distributed variable with log link function to estimate relative risk. An exchangeable working correlation was specified to account for repeated measurements on participants over time."		
**12a-i) Imputation techniques to deal with attrition / missing values**		
Imputation was not used in this study.		
**12b) CONSORT: Methods for additional analyses, such as subgroup analyses and adjusted analyses**		
"The baseline outcomes were included in the model due to baseline imbalances in the outcomes between the groups."		
RESULTS		
**13a) CONSORT: For each group, the numbers of participants who were randomly assigned, received intended treatment, and were analysed for the primary outcome**		
See CONSORT diagram, Figure 1.		
**13b) CONSORT: For each group, losses and exclusions after randomisation, together with reasons**		
See CONSORT diagram, Figure 1.		
**13b-i) Attrition diagram**		
See CONSORT diagram, Figure 1.		
**14a) CONSORT: Dates defining the periods of recruitment and follow-up**		
"Study enrollment began in December of 2016 and all follow-up visits were completed by October of 2020."		
**14a-i) Indicate if critical “secular events” fell into the study period**		
No secular events impacted this study.		
**14b) CONSORT: Why the trial ended or was stopped (early)**		
This is not applicable to this study as it was not stopped early.		
**15) CONSORT: A table showing baseline demographic and clinical characteristics for each group**		
See Table 1 of the manuscript.		
**15-i) Report demographics associated with digital divide issues**		
See Table 1 of the manuscript.		
**16a) CONSORT: For each group, number of participants (denominator) included in each analysis and whether the analysis was by original assigned groups**		
**16-i) Report multiple “denominators” and provide definitions**		
This study was intent-to-treat, thus the denominator is the same for the primary analyses.		
**16-ii) Primary analysis should be intent-to-treat**		
"The analysis used an intent-to-treat approach in which all randomized participants were included for analysis."		
**17a) CONSORT: For each primary and secondary outcome, results for each group, and the estimated effect size and its precision (such as 95% confidence interval)**		
See Tables 2-4 of the manuscript.		
**17a-i) Presentation of process outcomes such as metrics of use and intensity of use**		
The analysis for this study was intent-to-treat.		
**17b) CONSORT: For binary outcomes, presentation of both absolute and relative effect sizes is recommended**		
See Tables 2-4 of the manuscirpt.		
**18) CONSORT: Results of any other analyses performed, including subgroup analyses and adjusted analyses, distinguishing pre-specified from exploratory**		
See TAbles 2-4 of the manuscript.		
**18-i) Subgroup analysis of comparing only users**		
This analysis was intent-to-treat.		
**19) CONSORT: All important harms or unintended effects in each group**		
There were no unintended effects or consequences in this study.		
**19-i) Include privacy breaches, technical problems**		
There were no privacy breaches or unexpected/unintended incidents.		
**19-ii) Include qualitative feedback from participants or observations from staff/researchers**		

No qualitative feedback in the form of focus groups or interviews was collected in this study.		
DISCUSSION		
**20) CONSORT: Trial limitations, addressing sources of potential bias, imprecision, multiplicity of analyses**		
**20-i) Typical limitations in ehealth trials**		
"Given that this intervention was embedded within larger systems of care (i.e., placed within HIV testing clinics without the need for staffing), there would appear to be some value in considering further trials in this space. Further adaptation of the eSBI approach might result in greater impact."<br>"Our eSBI system can be considered a branch of the broader SBIRT model of care. This model has come under some criticism in recent years due to a lack of compelling evidence to show its impact as a primary prevention and intervention approach. Our eSBI system may have failed to achieve the intended goals of alcohol misuse reduction because of the brevity of the intervention, the lack of boosters or follow-up and/or the relatively simple design of the intervention."<br>"Given the instability of results for moderation of the intervention effect by symptoms of anxiety, future studies may need larger samples or be limited to the subpopulation of individuals with higher levels of anxiety to explore these effects more fully. "		
**21) CONSORT: Generalisability (external validity, applicability) of the trial findings**		
**21-i) Generalizability to other populations**		
"Future research may need to consider relational agents, digital interventions hybridized with human counselors or therapists, and interventions with boosters of some type. In addition, interventions that can address contextual factors (interpersonal, social and environmental) may help boost the effect of digital interventions like eSBI."		
**21-ii) Discuss if there were elements in the RCT that would be different in a routine application setting**		
This intervention was delivered as design for practice.		
**22) CONSORT: Interpretation consistent with results, balancing benefits and harms, and considering other relevant evidence**		
**22-i) Restate study questions and summarize the answers suggested by the data, starting with primary outcomes and process outcomes (use)**		
"In this study, we found no effect of the eSBI intervention on alcohol misuse, but there was evidence of an effect on sexual risk."		
**22-ii) Highlight unanswered new questions, suggest future research**		
"Despite the lack of findings for substance misuse, there was evidence of reduction in sexual risk in the intervention group in comparison to the control group for condomless anal insertive sex under the influence at 12-months. These analyses should be interpreted with caution as there were multiple secondary analyses done. Nevertheless, this finding is intriguing and should be the subject of additional study. "		
Other information		
**23) CONSORT: Registration number and name of trial registry**		
"Trial registration: ClinicalTrials.gov number, NCT02703116, registered March 9, 2016, https://clinicaltrials.gov/."		
**24) CONSORT: Where the full trial protocol can be accessed, if available**		
"Trial registration: ClinicalTrials.gov number, NCT02703116, registered March 9, 2016, https://clinicaltrials.gov/."		
**25) CONSORT: Sources of funding and other support (such as supply of drugs), role of funders**		
"This study is funded by the National Institute on Drug Abuse of the National Institutes of Health under Award Number R01-DA041071. Research reported in this publication was also supported by the National Institutes of Health's National Center for Advancing Translational Sciences, Grant Number UL1-TR001422. The content is solely the responsibility of the authors and does not necessarily represent the official views of the NIH."		
**X26-i) Comment on ethics committee approval**		
"The protocol was approved by the Institutional Review Boards of participating institutions with a waiver of parental permission for participation of minors (aged 16-17). All enrolled participants were consented prior to the start of any research activities, using an electronic consent form."		
**x26-ii) Outline informed consent procedures**		
"All enrolled participants were consented prior to the start of any research activities, using an electronic consent form."		
**X26-iii) Safety and security procedures**		
All participants were provided feedback on their level of alcohol use: "After completion of the AUDIT, participants received immediate feedback regarding their level of use."		
**X27-i) State the relation of the study team towards the system being evaluated**		
In terms of conflicts, "None declared"		
